# Arterial ischemic stroke in HIV: Defining and classifying etiology for research studies

**DOI:** 10.1212/NXI.0000000000000254

**Published:** 2016-06-30

**Authors:** Laura A. Benjamin, Alan Bryer, Sebastian Lucas, Alan Stanley, Theresa J. Allain, Elizabeth Joekes, Hedley Emsley, Ian Turnbull, Colin Downey, Cheng-Hock Toh, Kevin Brown, David Brown, Catherine Ison, Colin Smith, Elizabeth L. Corbett, Avindra Nath, Robert S. Heyderman, Myles D. Connor, Tom Solomon

**Affiliations:** From the Malawi-Liverpool-Wellcome Trust Clinical Research Programme (L.A.B., E.L.C., R.S.H.) and Department of Medicine (L.A.B., T.J.A.), University of Malawi College of Medicine, Blantyre; Institute of Infection and Global Health (L.A.B., H.E., T.S.), University of Liverpool; Walton Centre NHS Foundation Trust (L.A.B., T.S.), Liverpool, UK; Department of Medicine (A.B., A.S.), Division of Neurology, Groote Schuur Hospital, University of Cape Town, South Africa; Department of Histopathology (S.L.), St. Thomas Hospital, London; Radiology Department (E.J.) and Haematology Department (C.D., C.-H.T.), Royal Liverpool Hospital; Preston Hospital (H.E.); North Manchester General Hospital (I.T.); Virus Reference Department (K.B., D.B.) and Syphilis Reference Department (C.I.), Public Health England, London; Centre for Clinical Brain Sciences (C.S.) and Division of Clinical Neurosciences (M.D.C.), University of Edinburgh; Department of Clinical Research (E.L.C.), London School of Hygiene and Tropical Medicine, UK; National Institutes of Health (A.N.), Bethesda, MD; Division of Infection and Immunity (R.S.H.), University College London; NHS Borders (M.D.C.), Melrose, UK; School of Public Health (M.D.C.), University of the Witwatersrand, South Africa; and National Institute for Health Research Health Protection Research Unit in Emerging and Zoonotic Infections (T.S.), Liverpool, UK.

## Abstract

HIV infection, and potentially its treatment, increases the risk of an arterial ischemic stroke. Multiple etiologies and lack of clear case definitions inhibit progress in this field. Several etiologies, many treatable, are relevant to HIV-related stroke. To fully understand the mechanisms and the terminology used, a robust classification algorithm to help ascribe the various etiologies is needed. This consensus paper considers the strengths and limitations of current case definitions in the context of HIV infection. The case definitions for the major etiologies in HIV-related strokes were refined (e.g., varicella zoster vasculopathy and antiphospholipid syndrome) and in some instances new case definitions were described (e.g., HIV-associated vasculopathy). These case definitions provided a framework for an algorithm to help assign a final diagnosis, and help classify the subtypes of HIV etiology in ischemic stroke.

Stroke has become a prominent complication of HIV infection.^1,2^ Recent work suggests that the outcome of HIV-related stroke is also poor.^3^ A variety of mechanisms has been postulated, including opportunistic infections, cardio-thromboembolism, coagulopathy, and the incompletely understood HIV-associated vasculopathy.^4^ In addition, antiretroviral therapy may itself exacerbate ischemic stroke risk through several mechanisms.^2,4^ In some patients, multiple pathogenic processes may combine. Of the various types of stroke in people with HIV, arterial ischemic stroke affecting the brain is most frequently described, and so is the focus of this review.^4^

In the 1990s, approaches to studying the etiology of ischemic stroke advanced considerably with the development of the TOAST (Trial of Org 10172 in Acute Stroke Treatment) classification.^5^ However, the TOAST classification was derived in a setting where HIV infection was less prevalent; in these populations, approximately 75% of ischemic stroke patients are classified into 1 of 3 main categories (i.e., large artery atherosclerosis, small vessel disease [SVD], and cardio-thromboembolism).^6^ In contrast, in HIV-infected stroke populations, less than 50% fall into the 3 main categories.^7^ The higher proportion in the generic categories (i.e., “other determined,” and “undetermined”) is largely attributable to alternative causes and the occurrence of multiple etiologies in one individual. Without a reliable classification system, the study of HIV stroke, particularly its epidemiology and pathogenesis, will be inhibited.

Several types of vasculopathy have been described in individuals with HIV infection, including accelerated atherosclerosis, HIV-associated vasculitis, a nonatherosclerotic group with intimal hyperplasia but without atherosclerosis or vasculitis, and a group with radiologically and clinically defined SVD.^4^ While the larger vessel vasculopathies (e.g., the nonatherosclerotic group) may manifest as large artery stroke, SVD may be more important in HIV-associated neurocognitive disorders^8^; the pathogenesis of both is unclear. Greater consistency and accuracy across studies will be achieved by a standardized algorithm.

Recently, a ranked approach of applying diagnostic test results, which allows for different levels of evidence, was developed for defining the range of etiologies of encephalitis, which also facilitated new large prospective cohort studies.^9,10^ We therefore decided to adopt a similar approach to help classify the subtypes of HIV etiology in ischemic stroke. Our proposed algorithm is presented in figure 1.

**Figure 1.**
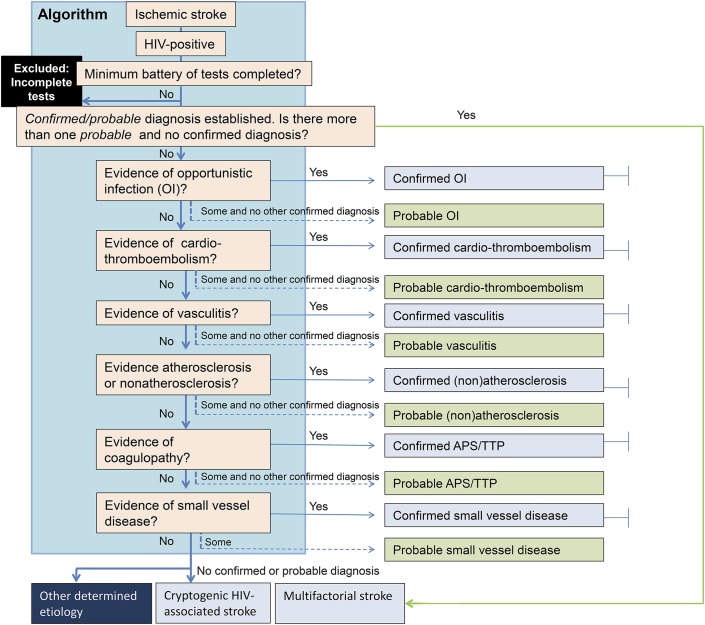
An algorithm to define the etiology of HIV-related ischemic stroke for research studies Minimum battery of tests include the following: HIV test, full blood count and blood film and urine dipstix assay, anticardiolipin antibodies, lupus anticoagulant, anti–β_2_-glycoprotein, hemoglobin for sickle cell disease, serum syphilis treponemal (immunoassay + agglutination test) and nontreponemal tests, chest x-ray, CSF—microscopy, biochemistry, India ink and acid fast bacilli stains, blood culture, tuberculosis culture, unenhanced CT, ECG, and carotid/vertebral duplex ultrasound and echocardiography. APS = antiphospholipid syndrome; OI = opportunistic infection; TTP = thrombotic thrombocytopenic purpura.

## METHODS

During the preparation of a recent review of HIV and stroke written by some of the present authors,^4^ it was clear that some case definitions needed refining, and more detailed consideration was needed in handling patients with multiple etiologies. We therefore identified experts in the fields of HIV infection and stroke and established a working group that included a broad range of relevant specialists.

The first step was to determine the main etiologies of interest for HIV-related arterial ischemic stroke; this was largely based on the classification described previously (table 1).^4^ Collectively, these etiologies should account for the majority of cases seen in HIV-related stroke.

**Table 1.**
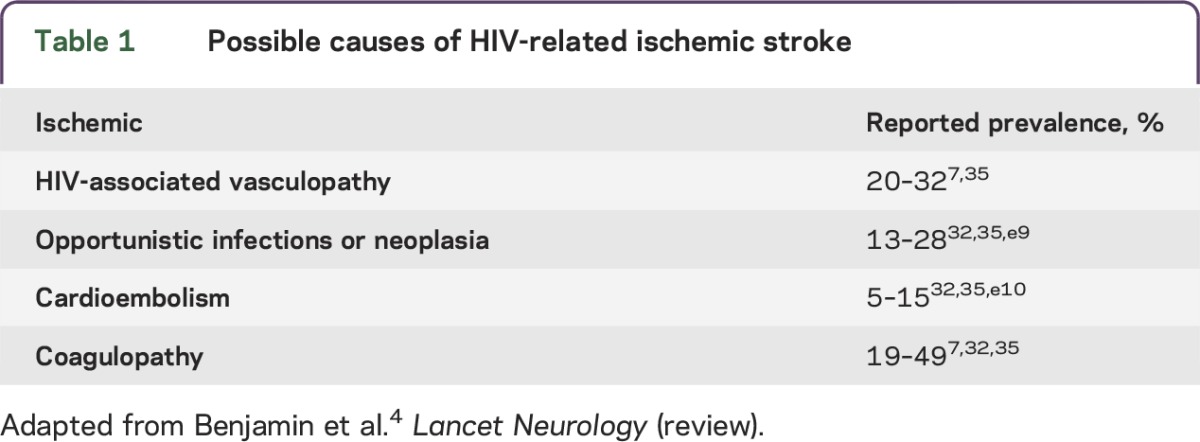
Possible causes of HIV-related ischemic stroke

A working template was developed to enable discussions between the group members. Ideas and proposals were discussed, deliberated, clarified, and modified based on e-mail input from all participants.

The diagnostic level of evidence used was not dissimilar from the TOAST classification or variations of it.^11–13^ For tests that had a “confirmed” diagnosis, there was Level A diagnostic evidence (i.e., direct demonstration by gold standard diagnostic tests or criteria) whereas for tests that determined a “probable” diagnosis, there was Level B diagnostic evidence (i.e., indirect evidence or less sensitive or specific tests or criteria). With the exception of a lumbar puncture for CSF analysis, our suggested tests form part of the usual workup for a stroke patient. Given that there are no contraindications, the consensus was that a lumbar puncture was justified to exclude infectious etiologies, irrespective of HIV stage and age of the individual.

We also defined a minimum investigation workup to help classify the different etiologies (table 2). The use of optimal tests further improved the level of evidence for causation. We then rationalized the ranking of the different etiologies to form an algorithm. The emerging roles of potential biomarkers were also considered. Our discussions formed the basis of this document.

**Table 2.**
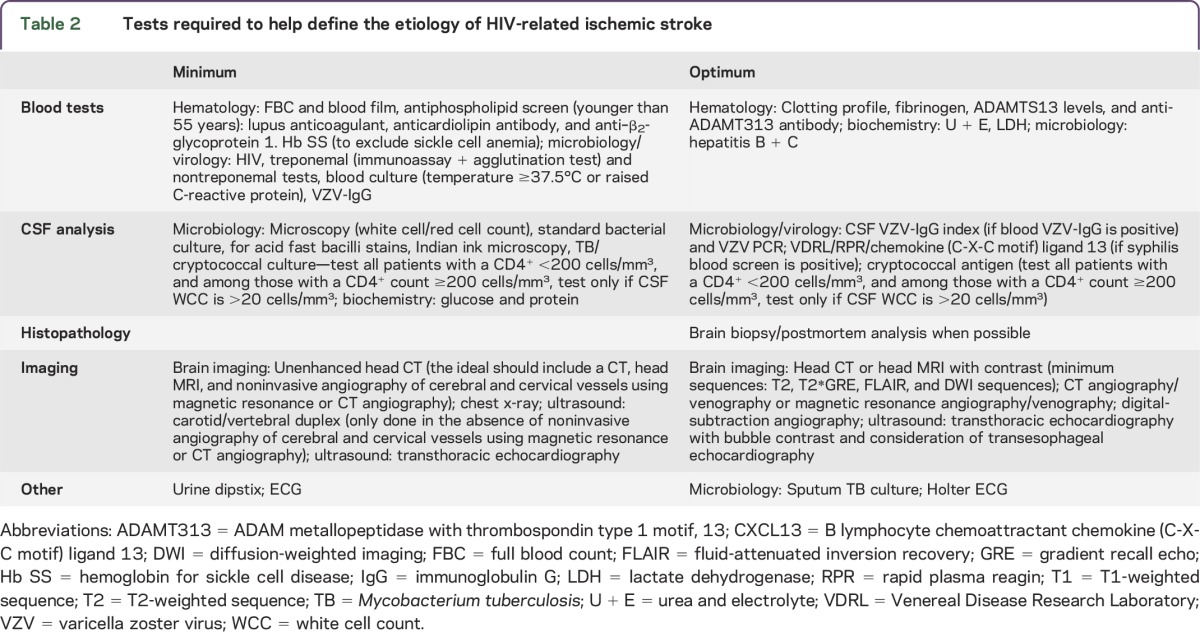
Tests required to help define the etiology of HIV-related ischemic stroke

## HANDLING MULTIPLE ETIOLOGIES IN HIV-RELATED STROKE: DEVELOPING THE ALGORITHM

### The entry point of this algorithm.

#### Arterial ischemic stroke.

This is based on the definition of the Stroke Council of the American Heart Association/American Stroke Association.^14^ Despite recent revisions to this case definition, those presenting with an insidious onset of cognitive deterioration associated with multifocal SVD on brain imaging, may still be overlooked.

#### HIV infection.

This is based on a positive antibody test (HIV enzyme immunoassay or rapid HIV antibody test) and confirmed with Western blot, antigen test, or PCR. In resource-poor settings, confirmation is usually with a second antibody test using a different manufacturer system.

#### A complete “minimum” assessment.

All patients should have at least the minimum set of investigations, as per our proposed algorithm (table 2). In better resourced settings, further investigations comprising the “optimum” set will improve the level of evidence to confirm a stroke etiology.

### The algorithm.

Having defined the criteria for the different etiologies of stroke in HIV, we developed an algorithm by which these etiologies would be considered (figure 1). For some, the causal role is clear, the disease mechanism relatively well described, and there are important treatment implications of making the diagnosis. For other putative causes, the evidence implicating them is less clear, and there are no direct treatment implications. For example, if a patient had evidence of both confirmed cardio-thromboembolic and atherosclerotic stroke, because the cardio-thromboembolic stroke has a high risk of recurrence and a poorer prognosis if untreated, this ranked higher.^15,16^ Hence, in deriving the algorithm, we adopted a hierarchy so that better established and treatable etiologies would be considered first. Thus, opportunistic infections were considered first, then cardio-thromboembolism, vasculitis, atherosclerotic or nonatherosclerotic vasculopathy, coagulopathies, and finally SVD. The evidence for these etiologies is discussed later.

Where there was evidence of multiple etiologies, a confirmed diagnosis would override any etiologies with evidence only for a probable diagnosis. If there was no confirmed diagnosis, then multiple probable diagnoses would be accepted. What has previously been the undetermined category has been reclassified in our algorithm as follows: (1) incomplete, defined by less than minimum tests completed; (2) multifactorial stroke, defined by 2 or more “probable” etiologies at the same level; and (3) cryptogenic stroke, defined by a complete minimum workup of tests but no etiology identified. This was further subdivided into (1) cryptogenic embolism (based on a radiology criterion),^17^ and (2) other cryptogenic—those not fulfilling the criteria for cryptogenic embolism.^17^ In the absence of invasive or noninvasive angiography, cryptogenic* stroke should be adopted, in which the asterisk denotes the absence of angiography.

## STRENGTHS AND LIMITATIONS OF CURRENT CASE DEFINITIONS FOR WELL-RECOGNIZED ETIOLOGIES IN HIV-RELATED ARTERIAL ISCHEMIC STROKE

### Opportunistic infections.

*Mycobacterium tuberculosis* (TB), *Cryptococcus*, varicella zoster virus (VZV), and syphilis are key infections recognized to cause stroke in people with HIV (table 3).^4^ Although they are more common in the immunosuppressed, coinfection (with HIV) in the immunocompetent is still pathogenic.

**Table 3.**
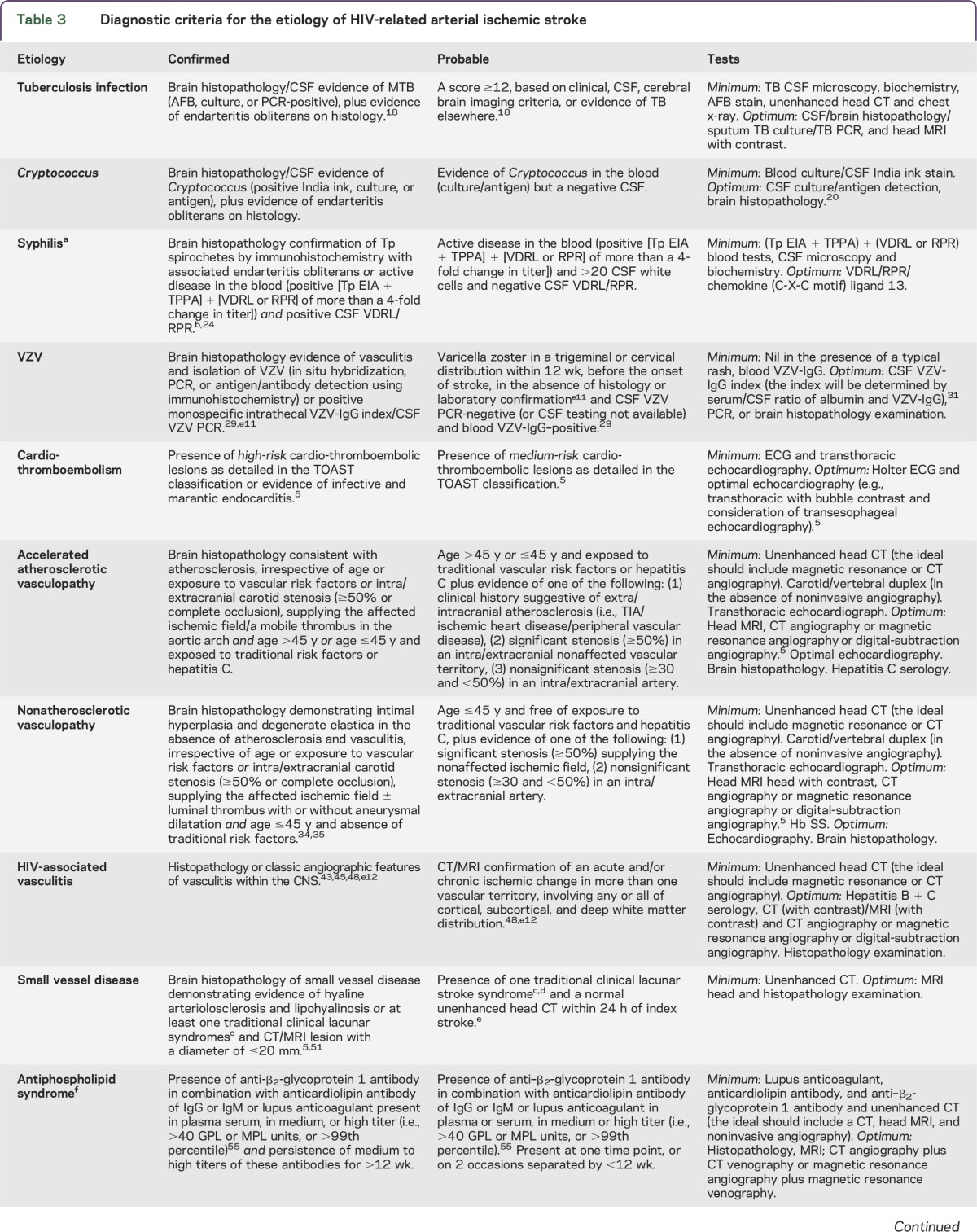

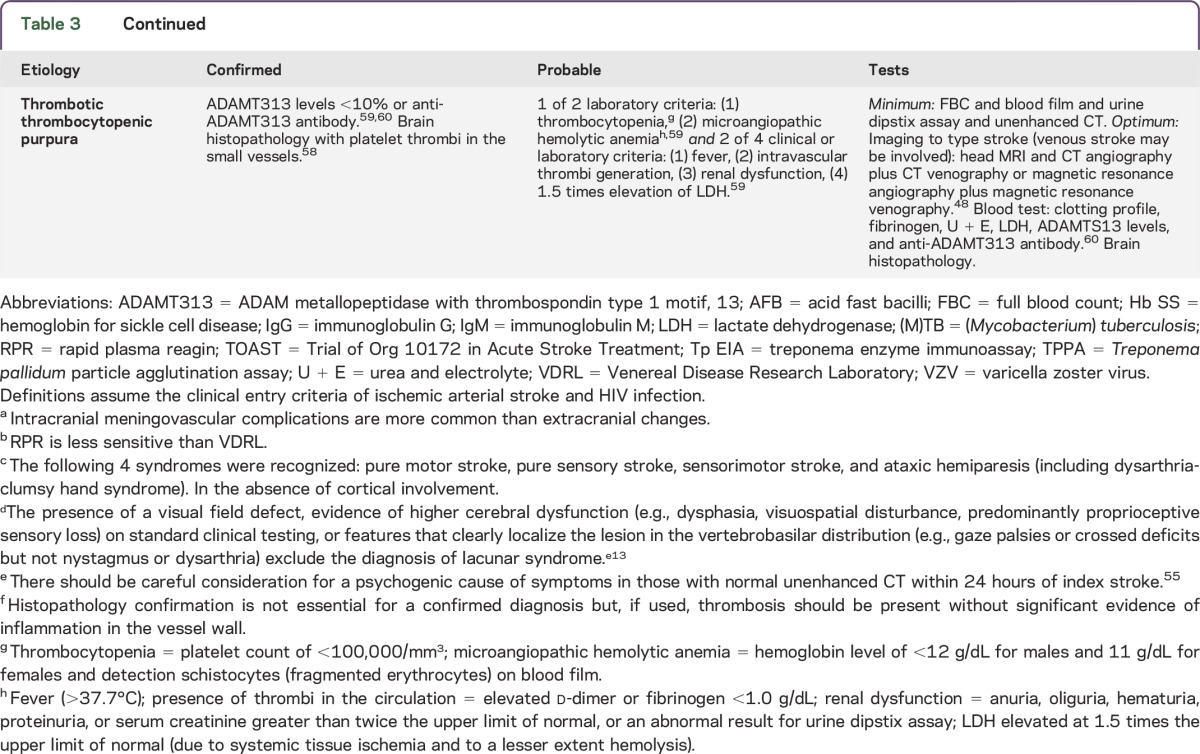
Diagnostic criteria for the etiology of HIV-related arterial ischemic stroke

#### Tuberculous meningitis.

TB in the CNS manifests in many ways, but the most common form, and that which most often leads to stroke, is tuberculous meningitis. The mechanism is through an obliterative endarteritis or vasospasm. The gold standard for diagnosing tuberculous meningitis is by isolating the bacillus in the brain or CSF. However, this is often difficult.^18^ Marais et al.^18^ have therefore developed a scoring algorithm for research studies based on clinical, CSF, imaging findings, and evidence of TB elsewhere.

#### *Cryptococcus*.

A stroke occurs in cryptococcal meningitis because of irritation of subarachnoid blood vessels, resulting in vasospasm or endarteritis from inflammation.^19^ Although culture of *Cryptococcus* from the CSF is the gold standard, in the context of symptomatic meningitis and a good sample volume of CSF, the sensitivity and specificity of India ink microscopy or cryptococcal antigen is greater than 90%, nearing 100% for cryptococcal antigen, and so this is often accepted.^20^ TB and cryptococcal infection arise more often in the immunosuppressed (i.e., CD4^+^ count <200 cells/mm^3^).^21,22^ Abnormal pleocytosis in the context of HIV typically accompanies these infections when the CD4^+^ count is ≥200 cells/mm^3^.^21,22^ We therefore recommend routine TB and cryptococcal testing in those with a CD4^+^ count <200 cells/mm^3^, and restricted testing to those with CSF pleocytosis at ≥200 cells/mm^3^.

#### Syphilis.

Stroke is a meningovascular complication of syphilis infection. A positive syphilis blood result can be a challenge to interpret in HIV populations. For example, HIV and antiphospholipid syndrome (APS) can give false-positive results using nontreponemal methods.^23^ Current recommendations suggest that a screening blood treponemal test (e.g., enzyme immunoassays) should be followed by an additional confirmatory treponemal test (e.g., *Treponema pallidum* particle agglutination assay), and if both of these are positive, a nontreponemal blood test (e.g., Venereal Disease Research Laboratory [VDRL] or rapid plasma reagin [RPR]) should be performed to determine whether this is active or previous disease.^24^ Having established active peripheral disease, neurosyphilis is diagnosed by a positive CSF VDRL or the less sensitive RPR.^25^ Because CSF VDRL/RPR can be negative during the early and late stages of neurosyphilis, CSF pleocytosis can be used to make a presumptive diagnosis. However, HIV is also associated with a CSF pleocytosis (≥5 cells/mm^3^), and some have suggested a cutoff of greater than 20 cells be used to diagnose neurosyphilis.^26^ CSF–fluorescence treponemal antibody test is sensitive for neurosyphilis but less specific than CSF VDRL/RPR in symptomatic cases (e.g., those with stroke); this could be useful in ruling out neurosyphilis, especially in asymptomatic cases but its utility in the context of HIV infection remains unclear.^26^ An additional new approach that has been validated in an HIV population, and may prove useful in the future, is CSF CXCL13 (B cell chemoattractant; chemokine [C-X-C motif] ligand 13).^27^

#### Varicella zoster virus.

The diagnosis of VZV vasculopathy is currently based on a positive CSF VZV–immunoglobulin G (IgG) or PCR.^28,29^ Although most hospitals rely on CSF VZV PCR, its use is limited by its lower sensitivity in stroke syndromes.^29^ HIV infection can impair the blood–brain barrier; as a result, blood VZV-IgG may leak into the CSF.^30^ Therefore, to overcome this limitation, diagnosis of VZV CNS vasculopathy should ideally involve calculating the VZV-antibody index.^30,31^ Furthermore, because other illnesses, including HIV, can cause a polyspecific intrathecal immune response, calculating the IgG antibody index for other viruses (e.g., rubella, measles, or herpes simplex [HSV]) will help to confirm the presence or absence of a monospecific antibody response to VZV-IgG.^31^ In the classic VZV stroke syndrome, delayed contralateral hemiparesis typically presents several weeks after acute herpes zoster ophthalmicus infection.^29^ We acknowledge that until intrathecal VZV-antibody testing becomes widely available, the diagnosis of VZV vasculopathy will largely be based on CSF PCR and/or the clinical features of stroke following a history of zoster rash in a corresponding distribution.^29^

#### Other infections.

HIV could potentially increase the rate of other CNS infections, such as HSV-1, cytomegalovirus, Epstein-Barr virus, toxoplasma, and fungal infections (e.g., aspergillosis, nocardiosis, and histoplasmosis), and these are potentially associated with stroke. However, there is a limited description in the literature of stroke directly related to these infections, suggesting that they may be less important.^7,32^

### Cardio-thromboembolism.

The criteria for cardio-thromboembolism are based largely on the TOAST classification.^5^ We however modified this to also include marantic (noninflammatory and nonbacterial) endocarditis in the high-risk group.^33^

### HIV-associated vasculopathy.

**Figure 2.**
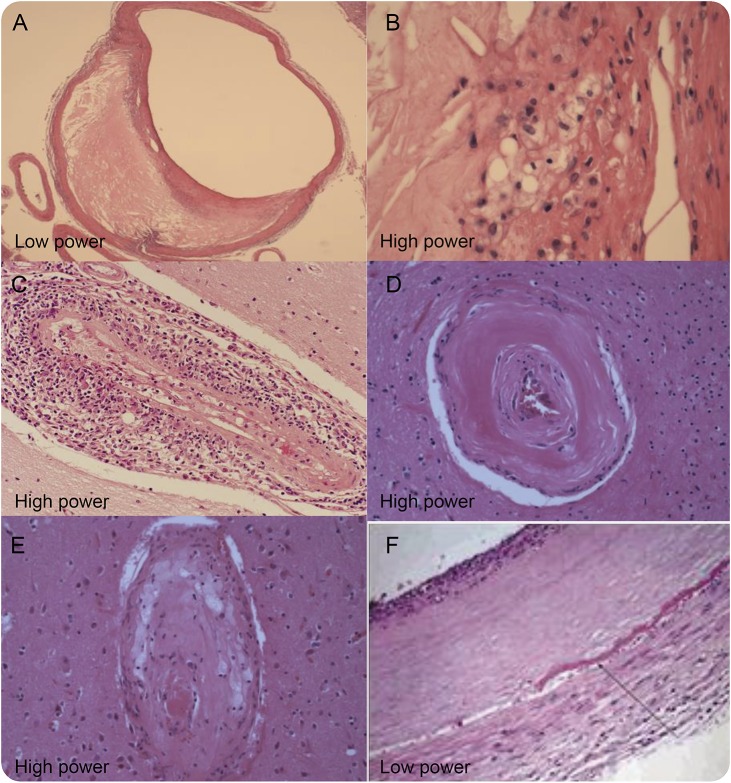
Different pathologic description of vasculopathy associated with HIV infection (A, B) Atherosclerotic vasculopathy. (C) HIV-associated vasculitis. (D, arteriolosclerosis; E, lipohyalinosis) Small vessel disease. (F) Nonatherosclerotic vasculopathy. (A–E) From personal archives. (F) Reproduced with permission from the *Archives of Neurology*. 2006. 63: 1640–1642. Copyright© 2006 American Medical Association. All rights reserved.^34^

HIV-associated vasculopathy was initially assigned to a pathologic phenotype with vessel wall abnormality in the absence of atherosclerosis or vasculitis.^34,35^ However, we found that vasculopathy, defined as intimal hyperplasia more than expected for age, also encompassed other pathologic phenotypes of stroke found in HIV infection, thus creating confusion in the literature. Examples included atherosclerosis, HIV-associated vasculitis, and SVD. In our recent review, we were deliberately inclusive by defining HIV-associated vasculopathy as an abnormality of the cerebral blood vessels that results directly or indirectly from HIV infection but excluding vasculitis due to opportunistic infections (figure 2).^4^ Each of the recognized phenotypes will now be discussed in turn.

#### Accelerated atherosclerotic vasculopathy.

Atherosclerosis is pathologically defined by plaque formation, consisting of foam cells, a lipid core, and a fibrous cap.^36^ It predominately affects large- to medium-sized vessels, and increases with age and exposure to traditional and emerging vascular risk factors such as hypertension, diabetes, smoking, high cholesterol, and hepatitis C.^37,38^ Diagnosis is largely based on radiologic evidence, characterized by significant (>50%) stenosis of the intra/extracranial artery (or its main branches) supplying the affected vascular field.^5^ Atherosclerosis is accelerated in HIV populations, occurring up to 2 decades earlier than expected.^39^ Chronic inflammation caused by incomplete suppression of HIV or dyslipidemia associated with some HIV treatments is believed to be the underlying mechanism.^4^

#### Nonatherosclerotic vasculopathy.

Nonatherosclerotic stroke also affects large- to medium-sized vessels; intimal hyperplasia that can progress to a stenotic or aneurysmal lesion is characteristic.^34,35^ Of note, in this condition, there is no evidence of atherosclerosis or vasculitis.^34,35,40^ After repeated damage to the vascular endothelium by HIV and/or its viral particles, it is conceivable that vessel wall remodeling arises; this has been described in a recent histopathology study.^40^ The vasculopathic changes are not dissimilar to observations seen in sickle cell disease in which repeated damage also occurs.^41^ Radiologic diagnosis is usually relied on but this does not distinguish nonatherosclerotic from atherosclerotic vasculopathy. However, young age of onset and the absence of traditional vascular risk factors do discriminate.^34,35^ Clinical conditions associated with atherosclerotic disease such as previous TIA, ischemic heart disease, or peripheral vascular disease are seldom reported in this group. In time, our understanding will expand to determine whether nonatherosclerotic vasculopathy is a precursor of atherosclerosis, a spectrum of HIV-associated vasculitis, or an independent process.

#### HIV-associated vasculitis.

The association of HIV and cerebral vasculitis has been reported in a few case series.^42–45^ It affects vessels of all sizes. Vasculitis can either be caused by infection, where direct invasion of the pathogen leads to proliferation and inflammation of the vessel wall (as is seen, for example, in VZV), or by noninfectious immune-mediated mechanisms (as seen in hepatitis B and polyarteritis nodosa).^46,47^ Although a pathogen may be indirectly involved in the latter, this type of vasculitis does not have direct vessel wall invasion by the pathogen. Thus far, histopathologic studies of HIV-associated cerebral vasculitis have failed to demonstrate HIV in the vessel wall, suggesting an immune-mediated mechanism.^4^ A temporal relationship between starting HIV treatment and the occurrence of a stroke might also point to an immune reconstitution syndrome, with HIV infection being an important precipitant of vasculitis.^2^

HIV-associated vasculitis is characterized by a single organ vasculitis (i.e., limited to the brain).^46^ Diagnosis usually involves identifying clinical and radiologic features and excluding an alternative cause. Causes of cerebral vasculitis frequently found in HIV infection include opportunistic infections (VZV, syphilis, *Cryptococcus*, TB) and APS; these have been discussed elsewhere in the article. Hepatitis B may coexist with HIV and should also be screened for.

There are rarer causes of vasculitis that are independent of HIV infection and only screened for in the presence of high clinical suspicion; examples include neoplasia (e.g., lung cancer), infections (e.g., neuroborreliosis), and inflammatory disorders (e.g., Behçet disease and sarcoid granulomatosis).^48^ For the purpose of this consensus report, we do not recommend that these are screened routinely unless there is a strong clinical suspicion.

#### Small vessel disease.

As a term, SVD covers many pathologies; the main causes described are associated with chronic hypertension, hyaline arteriolosclerosis and lipohyalinosis, and cerebral amyloid angiopathy.^49^ In HIV infection, autopsy series have demonstrated several pathologic characteristics consistent with hyaline arteriolosclerosis and lipohyalinosis.^50^ Protease-based antiretroviral therapy may also be implicated in the pathogenesis of SVD.^8^ The diagnosis of HIV-related SVD is largely based on radiology criteria^51^; this defines SVD as causing a recent infarct of less than 20 mm in the appropriate clinical context. In reality, this may not discriminate medium to small vessel disease from true SVD.

### Coagulopathy.

Although some studies have reported a high prevalence of coagulopathy in HIV-related ischemic stroke, the causal evidence for arterial-related coagulopathy, namely, APS and thrombotic thrombocytopenic purpura (TTP), is limited.^4,7,32,35,52,53^ Other coagulopathies such as protein C and S deficiency occur in HIV infection but are associated with venous and not arterial strokes.

#### Antiphospholipid syndrome.

More than 20% of APS cases present as a stroke.^54^ The revised classification criteria for APS (2006) require both clinical (i.e., ischemic stroke) and laboratory criteria for diagnosis (i.e., a positive anti–β_2_-glycoprotein I [anti-β_2_GP1] or anticardiolipin antibodies [ACL] or lupus anticoagulant [LA] detected in the blood and persisting for >12 weeks).^55^

There is some evidence of the usefulness of these criteria in diagnosing stroke caused by APS. Anti-β_2_GP1 and LA are strongly associated with stroke.^56^ However, the evidence for ACL as a predictor of APS and stroke is conflicting.^56,57^ Furthermore, HIV is associated with ACL and LA but not anti-β_2_GP1.^52^ As anti-β_2_GP1 is specific for stroke in HIV populations, the consensus was to refine the laboratory definition to include the detection of anti-β_2_GP1 in combination with ACL or LA. Because APS is found mostly in young populations, we have recommended testing only in those younger than 55 years.^54^

#### Thrombotic thrombocytopenic purpura.

TTP is a blood disorder that causes microscopic clots in the small vessels.^58^ HIV may be a direct precipitant of TTP through damage of vascular endothelial cells resulting in dysfunction, localized thrombin generation, and consumption of ADAMTS13 (a metalloprotease enzyme that cleaves von Willebrand factor).^59^ Diagnosis of TTP requires 2 major criteria (e.g., thrombocytopenia, microangiopathic hemolytic anemia, and neurologic signs) and at least 2 minor criteria (fever, renal dysfunction, presence of thrombi in the circulation, and elevated lactate dehydrogenase). Deficiency or antibody against ADAMTS13 confirms the diagnosis of TTP and helps to discriminate thrombocytopenia, microangiopathic hemolytic anemia occurring independently from TTP.^59,60^ Although testing for ADAMTS13 is not routine in clinical laboratories, it is necessary to confirm TTP in the research setting. In the context of HIV-associated stroke, this TTP classification was thought to be appropriate without modification.

### Other etiologies.

This list of etiologies is not exhaustive; there are other plausible mechanisms in HIV-related stroke but minimal evidence in the literature to support an association (e.g., systemic vasculitides, extra/intracranial arterial dissection, hyperviscosity syndrome, and other causes in an aging HIV population). There are also etiologies described in young populations that may co-occur in individuals with HIV infection (e.g., hereditary causes of stroke and drug-induced vasculopathy). A thorough clinical history and examination will direct additional investigations for these rarer causes of stroke. Future research studies or descriptive publications should provide as much clinical and radiologic information as possible to aid our understanding.

### Stroke mimics.

Approximately 15% of patients presenting with an acute focal neurologic deficit will have a stroke mimic, in HIV endemic populations.^2^ Some infections that can lead to a stroke can also mimic stroke. Mimics include toxoplasma infection, progressive multifocal leukoencephalopathy, viral encephalitides (e.g., HIV, HSV-1, cytomegalovirus), fungal infections (e.g., cryptococcoma), lymphoma, tuberculoma, and HIV-associated tumefactive demyelination.^e1–e3^ The selection of appropriate imaging (ideally a head MRI) is essential for the exclusion of these stroke mimics.

### Biomarkers in cerebrovascular disease.

Several promising candidate biomarkers associated with vascular disease have been identified (e.g., MCP-1, sCD14, cerebral vasoreactivity).^e4,e5^ Although some biomarkers, such as tumor necrosis factor receptors 1 and 2, interleukin 6, and highly sensitive C-reactive protein, are elevated in HIV infection and predict non-AIDs complications, the association with vascular disease subtype remains largely uncertain.^e6,e7^ Such biomarkers could prove to be valuable in the future, especially in the context of HIV-associated vasculopathy.

## DISCUSSION

In this consensus statement, we have refined several preexisting case definitions for causes of stroke in people with HIV and developed new definitions for HIV-associated vasculopathy. We also produced an algorithm to assign a diagnosis when multiple etiologies arise.

Stroke in low- to middle-income regions is increasing. In many of these regions, HIV is prevalent, and younger populations are more likely to have infectious causes of stroke. We will only begin to understand the different mechanisms in HIV-related stroke if we have robust case definitions and diagnostic algorithms.

Our vertical algorithm adopts a hierarchical approach, giving greater weight to those etiologies that are well established and or for which there are treatment implications. We also defined a “resource-sensitive” minimum battery of investigations to help determine such etiologies. Most research studies will benefit from this approach. Validation in prospective cohorts will help to determine its utility in clinical practice.

Recent work has shown an association between HIV infection and intracerebral hemorrhage.^e8^ Future algorithms may need to also incorporate this pathologic type of stroke.

An important limitation is relying on head CT alone. The challenge is with classifying subtypes of HIV-associated vasculopathy when the pathology is intracranial and therefore not captured by duplex carotid Doppler. Although infrequent, it could lead to cases being misclassified as cryptogenic stroke. There is also the risk of misclassifying SVD; this could be minimized by more than one radiologist reviewing the images and a consensus determined.

We think our pragmatic approach to classifying ischemic stroke would allow more refined systematic investigations of subtype-specific etiologies and therapies.

## Supplementary Material

Data Supplement

## GLOSSARY

ACL: anticardiolipin antibodies
anti-β2GP1: anti–β2-glycoprotein I
APS: antiphospholipid syndrome
HSV: herpes simplex virus
IgG: immunoglobulin G
LA: lupus anticoagulant
RPR: rapid plasma reagin
SVD: small vessel disease
TB: tuberculosis
TOAST: Trial of Org 10172 in Acute Stroke Treatment
TTP: thrombotic thrombocytopenic purpura
VDRL: Venereal Disease Research Laboratory
VZV: varicella zoster virus
