# Supplementary material for: Arterial ischemic stroke in HIV: Defining and classifying etiology for research studies
Source: Neurol Neuroimmunol Neuroinflamm. 2016 Jun 30;3(4):e254. doi: 10.1212/NXI.0000000000000254 (PMC4929887; doi:10.1212/NXI.0000000000000254)
Supplement: Data Supplement [file supp_3_4_e254__index.html]

Data Supplement 

# Arterial ischemic stroke in HIV

## Data Supplement

**Files in this Data Supplement:**

- Online References - Microsoft Word file
