# Supplementary material for: Arterial ischemic stroke in HIV: Defining and classifying etiology for research studies
Source: Neurol Neuroimmunol Neuroinflamm. 2016 Jun 30;3(4):e254. doi: 10.1212/NXI.0000000000000254 (PMC4929887; doi:10.1212/NXI.0000000000000254)
Supplement: Data Supplement [file supp_3.4.e254_Final_Online_References.docx]

e1. Bingham R, Ahmed N, Rangi P, Johnson M, Tyrer M, Green J. HIV encephalitis despite suppressed viraemia: a case of compartmentalized viral escape. Int J STD AIDS 2011;22:608-609.

e2. Uriel A, Stow R, Johnson L, et al. Tumefactive demyelination-an unusual neurological presentation of HIV. Clin Infect Dis 2010;51:1217-1220.

e3. Benjamin LA, Joekes E, Das K, Beeching NJ, Wilkins E, Solomon T. Diagnostic accuracy of the Recognition of Stroke in the Emergency Room (ROSIER) score and CT brain in an HIV population. J Infect 2013.

e4. Gonzalez-Quesada C, Frangogiannis NG. Monocyte chemoattractant protein-1/CCL2 as a biomarker in acute coronary syndromes. Curr Atheroscler Rep 2009;11:131-138.

e5. Chow FC, Boscardin WJ, Mills C, et al. Cerebral vasoreactivity is impaired in treated, virally suppressed HIV-infected individuals. AIDS 2016;30:45-55.

e6. Guzman-Fulgencio M, Medrano J, Rallon N, et al. Soluble markers of inflammation are associated with Framingham scores in HIV-infected patients on suppressive antiretroviral therapy. J Infect 2011;63:382-390.

e7. McComsey GA, Kitch D, Sax PE, et al. Associations of inflammatory markers with AIDS and non-AIDS clinical events after initiation of antiretroviral therapy: AIDS clinical trials group A5224s, a substudy of ACTG A5202. J Acquir Immune Defic Syndr 2014;65:167-174.

e8. Chow FC, He W, Bacchetti P, et al. Elevated rates of intracerebral hemorrhage in individuals from a US clinical care HIV cohort. Neurology 2014;83:1705-1711.

e9. Patel VB, Sacoor Z, Francis P, Bill PL, Bhigjee AI, Connolly C. Ischemic stroke in young HIV-positive patients in Kwazulu-Natal, South Africa. Neurology 2005;65:759-761.

e10. Hoffmann M. Stroke in the young in South Africa--an analysis of 320 patients. S Afr Med J 2000;90:1226-1237.

e11. Gilden D, Cohrs RJ, Mahalingam R, Nagel MA. Varicella zoster virus vasculopathies: diverse clinical manifestations, laboratory features, pathogenesis, and treatment. Lancet Neurol 2009;8:731-740.

e12. Kuker W. Cerebral vasculitis: imaging signs revisited. Neuroradiology 2007;49:471-479.

e13. Bamford J, Sandercock P, Jones L, Warlow C. The natural history of lacunar infarction: the Oxfordshire Community Stroke Project. Stroke 1987;18:545-551.
